# Need for Improved Methods to Collect and Present Spatial Epidemiologic Data for Vectorborne Diseases

**DOI:** 10.3201/eid1312.070211

**Published:** 2007-12

**Authors:** Lars Eisen, Rebecca J. Eisen

**Affiliations:** * Colorado State University, Fort Collins, Colorado, USA; † Centers for Disease Control and Prevention, Fort Collins, Colorado, USA

**Keywords:** Census tract, county, exposure site, incidence, spatial epidemiology, vector-borne disease, ZIP code, United States, perspective

## Abstract

Improved methods for collection and presentation of spatial epidemiologic data are needed for vectorborne diseases in the United States. Lack of reliable data for probable pathogen exposure site has emerged as a major obstacle to the development of predictive spatial risk models. Although plague case investigations can serve as a model for how to ideally generate needed information, this comprehensive approach is cost-prohibitive for more common and less severe diseases. New methods are urgently needed to determine probable pathogen exposure sites that will yield reliable results while taking into account economic and time constraints of the public health system and attending physicians. Recent data demonstrate the need for a change from use of the county spatial unit for presentation of incidence of vectorborne diseases to more precise ZIP code or census tract scales. Such fine-scale spatial risk patterns can be communicated to the public and medical community through Web-mapping approaches.

## Abstract 2

These methods will improve capability for development of spatial risk models for vectorborne diseases in the United States.

Risk for human exposure to arthropod vectors and their associated pathogens (e.g., the tickborne Lyme disease spirochete *Borrelia burgdorferi*, fleaborne plague bacterium *Yersinia pestis*, and mosquitoborne West Nile virus [WNV]) is spatially highly heterogeneous in the United States (*1**–**16*). This concept can be exemplified by the spatial distributions of plague cases and areas with high projected plague risk in Arizona, New Mexico, Utah, and Colorado (Figure 1) and incidence of endemic Lyme disease in California (Figure 2) (*8**,**9*). Such heterogeneity in spatial risk patterns results in part from variability in environmental suitability for the vectors, especially with regard to climate factors and habitat type, and abundance of vertebrate hosts or pathogen reservoirs (*17**–**21*). Three examples can illustrate this point. First, exposure to *Ixodes pacificus* nymphs, which serve as primary vectors of *B*. *burgdorferi* in California, is largely restricted to dense woodlands with a ground cover dominated by leaf litter and lacking emergent vegetation (*22**,**23*). Moreover, density of nymphs and *B*. *burgdorferi*–infected nymphs differs between different woodland types; oak woodlands show a greater risk for exposure to the Lyme disease agent than redwood habitats (*6**,**7*). These differences represent crucial knowledge in assessment of probable pathogen exposure sites for Lyme disease cases in California.

**Figure 1:**
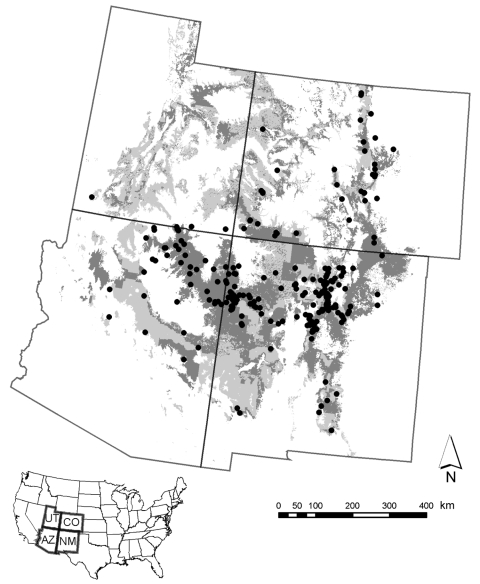
Areas predicted by a model based on peridomestically acquired plague cases from 1957 through 2004 to pose high risk to humans in the Four Corners Region (Arizona, Colorado, New Mexico, and Utah) are depicted in light gray. Those high-risk areas on privately or tribally owned land are shown in dark gray. Black circles represent locations of peridomestically acquired human plague cases. States comprising the Four Corners Region are shown within the United States in the inset. Reprinted with permission of the Journal of Medical Entomology from Eisen et al. (*9*).

**Figure 2:**
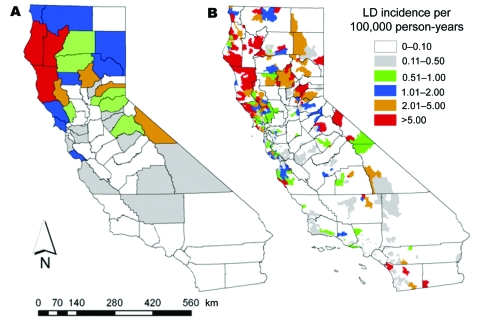
Comparison of spatial distributions of areas of California with different incidences of endemic Lyme disease (LD), 1993–2005, when calculated by A) the county spatial unit and B) the 5-digit ZIP code spatial unit. Adapted from a figure published in the American Journal of Tropical Medicine and Hygiene by Eisen et al. (*8*).

Second, spatial patterns of distribution and abundance of the mosquito *Culex tarsalis*, which is considered the primary vector to humans of WNV in the western United States, are related to both climatic conditions and suitable mosquito larval habitats (*13**,**14**,**24**–**28*). In Colorado, which had a WNV disease outbreak with 2,947 reported human cases in 2003, the spatial pattern of abundance of *Cx*. *tarsalis* is highly heterogeneous. The mosquito occurs commonly only at lower elevations <1,800 m (*24**,**25*), and its presence in the semiarid plains landscape characteristic of eastern Colorado is strongly correlated with availability of water sources (natural or resulting from irrigation) for the immature aquatic mosquito life stages. Assessments of probable WNV exposure sites in Colorado are complicated by inadequate knowledge of the fine-scale spatial distributions of key *Culex* spp. WNV vectors (*Cx*. *pipiens*, *Cx*. *tarsalis*) and the fact that people commonly are bitten by other mosquitoes in areas where these vectors and WNV are absent (e.g., the high mountains in central Colorado).

Third, human plague cases in the southwestern United States are closely associated with ecotonal piñon-juniper habitat and elevation (*9*). The etiologic agent of plague is transmitted primarily by flea bite, and human cases are typically associated with epizootic activity, which most commonly occurs in clearly defined habitat types and under climatic conditions favoring build-up of dense rodent and flea populations (*17**,**18**,**29**,**30*). Exhaustive plague case investigations by state health agencies or the Centers for Disease Control and Prevention (CDC) ensure reliable assessments of probable exposure sites for *Y*. *pestis* in the United States.

## Improving Data for Probable Pathogen Exposure Site

Over the past decade, advances in geographic information system technology have facilitated the development of predictive spatial models for risk for exposure to key vectors and pathogens in the United States (*1**,**3**,**5**,**7**–**12**,**16*). However, lack of reliable data for probable pathogen exposure sites has emerged as a major obstacle to the development of spatial epidemiologic and ecoepidemiologic models. In the United States, comprehensive case investigations by teams that include epidemiologists and vector ecologists and the determination of probable pathogen exposure sites are routinely conducted only for plague. Although the plague case investigation can serve as a model for how to ideally generate needed information for probable pathogen exposure sites, this exhaustive approach is cost-prohibitive for more common and less severe diseases such as Lyme disease and WNV disease. Unless the public health system is willing to invest funds needed to conduct comprehensive case investigations for a given vectorborne disease, determinations of probable pathogen exposure sites will remain the responsibility of the attending physician. Physicians may not be willing to spend the time required to obtain extensive patient travel histories to determine probable pathogen exposure site, and their lack of training in vector ecology impedes their ability to collect relevant information.

To solve this problem, new methods are needed to determine probable pathogen exposure site that yield reliable results while taking into account economic and time constraints of the public health system and attending physicians. These methods could, for example, include sets of standardized questions developed by CDC and tailored to a given vectorborne disease. A critical minimal need includes a basic assessment of whether pathogen exposure likely occurred in 1) the peridomestic environment, 2) outside the peridomestic environment but within the county of residence, or 3) outside the county of residence. The role of this issue for spatial epidemiologic modeling was demonstrated by our recent study of Lyme disease in California where reexamination of Lyme disease case files from 1993 through 2005 showed that 27% of the 1,325 case-patients had likely been exposed to the pathogen outside the county of residence (*8*). Other possible approaches include the point-radius method for georeferencing of probable pathogen exposure sites on the basis of patient activity space patterns (*31*). Research is needed to determine the value and feasibility of implementing these or other methods into routine public health activities.

## Spatial Unit for Calculation and Presentation of Incidence of Vectorborne Disease

**Figure 3:**
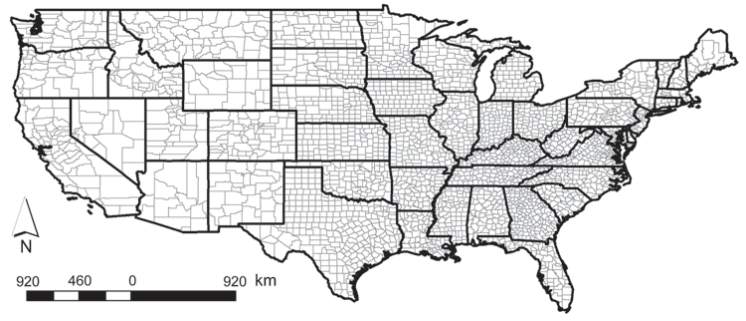
State and county boundaries within the contiguous United States. Note the increasing size of counties from east to west.

CDC and individual state health agencies routinely use county as the spatial unit for calculating and presenting incidence of vectorborne disease. The main problem with using county-based incidences for vectorborne diseases is that incidences calculated at this relatively crude spatial scale obscure fine-scale risk patterns commonly occurring within a county. This is especially problematic in the western United States, where many counties cover extensive areas (Figure 3) and encompass considerable ecologic and climatic variability. It was therefore not surprising that Eisen et al. (*8*) found that calculation and presentation of incidence of endemic Lyme disease in California at the county spatial unit, relative to the 5-digit ZIP code spatial unit, served to obscure small, isolated high-risk areas in the southern part of the state and the spatial variability of risk within high-risk counties (Figure 2).

Knowledge of local areas and habitats representing risk for vector exposure can be a major component in a diagnosis of probable Lyme disease or plague because infected persons may be unaware of receiving a tick or flea bite (*32**–**34*). Such knowledge is crucial in areas of the United States where the disease in question occurs but is rare. For example, in the absence of a documented tick bite and without knowledge that there are local areas with risk for exposure to the Lyme disease agent, a physician may be unlikely to consider Lyme disease as a possible diagnosis unless the patient has visited some other area the physician perceives to pose risk for exposure to the Lyme disease agent. Recognizing heterogeneity in spatial risk patterns for plague is similarly critical because it will aid local public health workers in targeting education of healthcare providers and the public to areas with a high risk for exposure to the plague agent (*9**,**35*). Prevention and treatment guidelines are well established for plague, but outcome of infection is improved by early diagnosis followed by appropriate treatment with antimicrobial drugs (*36*).

The 2 primary options in a shift away from using the county spatial unit for vectorborne disease incidence calculations are 1) ZIP code/ZIP code tabulation area and 2) census tract. There are pros and cons for each option. The 5-digit ZIP code unit is convenient because information regarding ZIP code of home address is readily collected during a visit to a physician, and the public is well aware of their ZIP code of residence and therefore can make ready use of information in map formats for ZIP code–based risk patterns. Conversely, a recent publication (*37*) raised concerns regarding increasing use of ZIP codes/ZIP code tabulation areas in spatial analyses of epidemiologic data because of their lack of standardization and dynamic spatial structure.

The more permanent census tract spatial unit, which tends to be smaller than the 5-digit ZIP code unit in population centers but can be larger than the ZIP code unit in sparsely populated areas, is attractive because it has a more uniform population base (typically 1,500–8,000 persons) than the ZIP code unit and therefore is less prone to the problem of overestimation of disease incidence on the basis of a few cases among a low population base. As demonstrated for WNV disease in Colorado by the Colorado Department of Public Health and Environment, some state level agencies have already adopted the practice of using the census tract unit to present spatial patterns of vectorborne diseases (www.cdphe.state.co.us/dc/zoonosis/wnv). Research is needed to evaluate the relative benefits of using the ZIP code compared with the census tract unit for calculation and presentation of spatial patterns of different vectorborne diseases.

Finally, advances in geographic information system technology and the ever-increasing use of the Internet as a primary knowledge resource provide tremendous possibilities for disseminating information regarding spatially explicit risk for exposure to vectorborne pathogens. Using a Web-mapping approach, one could easily convert static maps for plague and Lyme disease (Figures 1, 2) into a Web-based information delivery system in which selecting a county of interest provides a closeup view of the county, showing risk patterns for labeled ZIP codes and the location of major roads, population centers, and heavily used recreation areas.

## Conclusions

New methods of determining probable pathogen exposure site that yield reliable results while taking into account economical and time constraints of the public health system are urgently needed to improve capability for developing predictive spatial risk models for vectorborne diseases in the United States. Recent data also demonstrate the need for a change from use of the crude county spatial unit for presentation of incidence of vectorborne diseases to finer ZIP code or census tract scales. Communication of such fine-scale spatial risk patterns to the public and medical community can be achieved through Web-mapping approaches.
